# Supplementary material for: Homomorphic ZW chromosomes in a wild strawberry show distinctive recombination heterogeneity but a small sex‐determining region
Source: New Phytol. 2016 Apr 22;211(4):1412–23. doi: 10.1111/nph.13983 (PMC5074332; doi:10.1111/nph.13983)
Supplement: Supplementary file 1 — Fig. S1 Haplotypes in the sex‐determining region (SDR) vicinity of Fragaria chiloensis. [file NPH-211-1412-s001.pdf]

New Phytologist

New Phytologist **Supporting Information**

Homomorphic ZW chromosomes in a wild strawberry show distinctive recombination heterogeneity but a small sex-determining region

Jacob A Tennessen, Rajanikanth Govindarajulu, Aaron Liston and Tia-Lynn Ashman

Article acceptance date: 21 March 2016

The following Supporting Information is available for this article:

Fig. S1 Haplotypes in the sex determining region (SDR) vicinity of *Fragaria chiloensis*.

Table S1 IDs and collection localities of plants unrelated to the parents of the crosses (see separate Excel file).

Table S2 Primers for amplicon sequencing (see separate Excel file).

Table S3 PCR conditions for all amplicons (see separate Excel file).

Table S4 Phenotypes and genotypes for all HM1×SAL3 offspring (see separate Excel file).

Table S5 Phenotypes and genotypes of EUR13×EUR3 and PTR14×PTR19 offspring (see separate Excel file).

Table S6 Positions of all markers in target capture linkage maps (see separate Excel file).

Table S7 List of 70 genes in the 280 kb window on chromosome Fvb6 matching the *F. chiloensis* SDR (see separate Excel file).

Table S8 Recombination rates for all linkage groups (see separate Excel file).

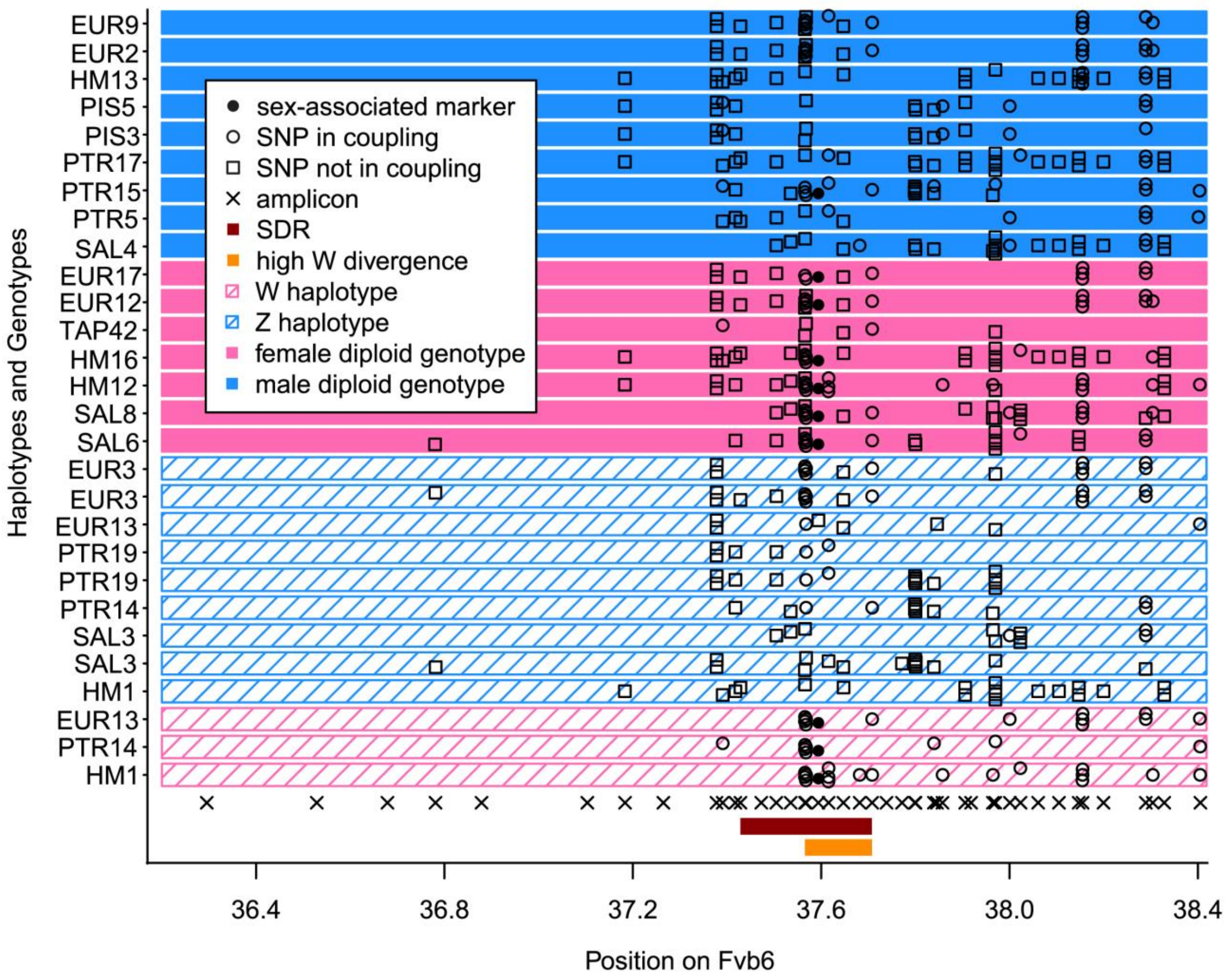

Fig. S1 Haplotypes in the sex determining region (SDR) vicinity of *Fragaria chiloensis*. We resolved twelve haplotypes, two from each of the parents of three crosses (diagonally shaded bars). For the additional unrelated plants, we did not phase haplotypes but instead show all single nucleotide polymorphisms (SNPs) present in each diploid genotype (solid bars). SNPs that mapped to VI-Av-m in at least one cross were observed from amplicons (indicated with X's), and are plotted with an x-axis position corresponding to physical position on reference genome chromosome Fvb6. Within a haplotype, SNPs are staggered vertically if more than one occurred in the same amplicon. SNPs are categorized based on whether they are ever observed in coupling with sex (circles) or not (squares). The sex-associated marker showing a correlation with sex across most plants is shown as a solid circle. A high density of SNPs in coupling with sex occurs in the SDR, especially in the 'high W divergence' region from 37.565-37.708Mb.
